# Translating the Genome into Drugs

**DOI:** 10.1021/acs.accounts.2c00791

**Published:** 2023-02-09

**Authors:** Anjali Dixit, Huda Barhoosh, Brian M. Paegel

**Affiliations:** ^†^Department of Pharmaceutical Sciences, ^‡^Department of Chemistry, and ^§^Department of Biomedical Engineering, University of California, Irvine, California 92697, United States

## Abstract

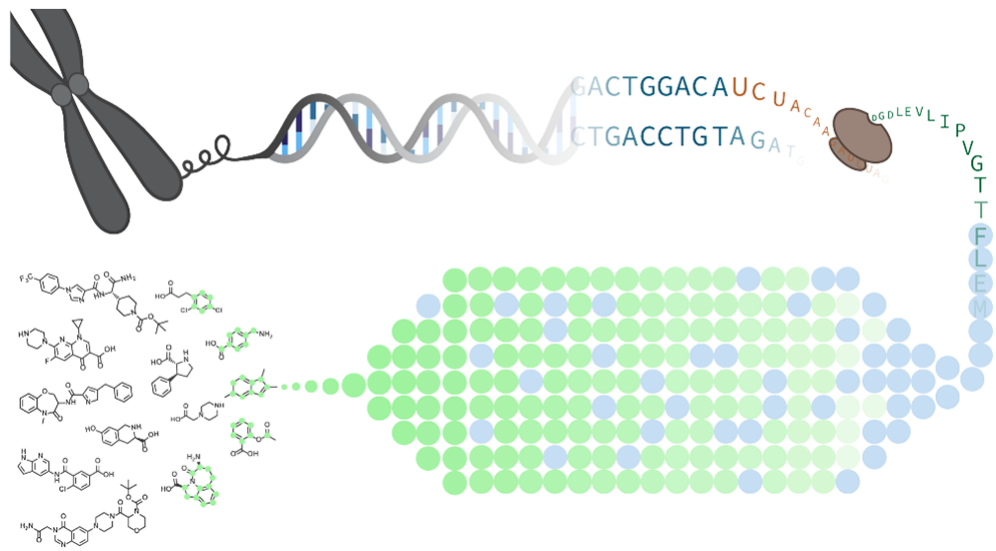

The Human Genome Project ultimately aimed to translate DNA sequence into drugs. With the draft in hand, the Molecular Libraries Program set out to prosecute all genome-encoded proteins for drug discovery with automated high-throughput screening (HTS). This ambitious vision remains unfulfilled, even while innovations in sequencing technology have fully democratized access to genome-scale sequencing. Why? While the central dogma of biology allows us to chart the entirety of cellular metabolism through sequencing, there is no direct coding for chemistry. The rules of base pairing that relate DNA gene to RNA transcript and amino acid sequence do not exist for relating small-molecule structure with macromolecular binding partners and subsequently cellular function. Obtaining such relationships genome-wide is unapproachable via state-of-the-art HTS, akin to attempting genome-wide association studies using turn-of-the-millennium Sanger DNA sequencing.

Our laboratory has been engaged in a multipronged technology development campaign to revolutionize molecular screening through miniaturization in pursuit of genome-scale drug discovery capabilities. The compound library was ripe for miniaturization: it clearly needed to become a consumable. We employed DNA-encoded library (DEL) synthesis principles in the development of solid-phase DELs prepared on microscopic beads, each harboring 100 fmol of a single library member and a DNA tag whose sequence describes the structure of the library member. Loading these DEL beads into 100 pL microfluidic droplets followed by online photocleavage, incubation, fluorescence-activated droplet sorting, and DNA sequencing of the sorted DEL beads reveals the chemical structures of bioactive compounds. This scalable library synthesis and screening platform has proven useful in several proof-of-concept projects involving current clinical targets.

Moving forward, we face the problem of druggability and proteome-scale assay development. Developing biochemical or cellular assays for all genome-encoded targets is not scalable and likely impossible as most proteins have ill-defined or unknown activity and may not function outside of their native contexts. These are the dark undruggable expanses, and charting them will require advanced synthesis and analytical technologies that can generalize probe discovery, irrespective of mature protein function, to fulfill the Genome Project’s vision of proteome-wide control of cellular pharmacology.

## Key References

- CochraneW. G.; MaloneM. L.; DangV. Q.; CavettV.; SatzA. L.; PaegelB. M.Activity-Based DNA-Encoded Library Screening. ACS Comb. Sci.2019, 21, 425–43510.1021/acscombsci.9b0003730884226PMC6786493(1)*In this first demonstration of activity-based DEL screening, a 67100-member DEL was screened for autotaxin (ATX) inhibitors and the results were compared with conventional affinity selection-based DEL analysis.*
- HacklerA. L.; FitzGeraldF. G.; DangV. Q.; SatzA. L.; PaegelB. M.Off-DNA DNA-Encoded Library Affinity Screening. ACS Comb. Sci.2020, 22, 25–3410.1021/acscombsci.9b0015331829554PMC6957756(2)*Off-DNA DEL screening was demonstrated using fluorescence polarization (FP) to detect competition binding between DEL members and FP probes, eliminating potential interference with the DNA encoding tag.*
- CochraneW. G.; FitzgeraldP. R.; PaegelB. M.Antibacterial Discovery via Phenotypic DNA-Encoded Library Screening. ACS Chem. Biol.2021, 16, 2752–275610.1021/acschembio.1c0071434806373PMC8688339(3)*Activity-based DEL technology was expanded to bacterial cell-based screening using a lawn bead diffusion assay; multiple cell-active hit structure families recapitulated known fluoroquinolone structure–activity relationships.*
- MacConnellA. B.; PaegelB. M.Poisson Statistics of Combinatorial Library Sampling Predict False Discovery Rates of Screening. ACS Comb. Sci.2017, 19, 524–53210.1021/acscombsci.7b0006128682059PMC5558193(4)*Monte Carlo simulations of screening experiments provided a theoretical framework for establishing a mathematical model of false discovery rates and parameters for optimizing activity-based DEL screening.*

## 1 Introduction

The human proteome as revealed through genome sequencing is a trove of potential targets for drug discovery. This was the original vision of NIH’s Molecular Libraries Program, which established molecular screening centers modeled after industrial high-throughput screening (HTS) platforms to translate the genome-encoded targets into drugs.^5^ Although enormously productive,^6^ the combined output of these centers (∼400 molecular probes) fell well short of the ∼20000 genome-encoded protein targets, let alone the astronomically wider diversity of the interactome. If the tools existed to discover molecules that modulate the entirety of the proteome, then how would this change our understanding of cellular pharmacology and the development of new medicines?^7^ Such technology would be revolutionary, analogous to the explosion in sequencing-based analyses that have unveiled cell-specific transcriptomic activity, the positions of all translating ribosomes, and even the three-dimensional structure of the genome itself. State-of-the-art drug discovery by HTS, by comparison, does not approach the extent to which genome-scale DNA sequencing technology has become accessible to researchers globally, but proteome-wide probe discovery capabilities will demand this level of distributed activity. Achieving this degree of technology distribution would minimally require miniaturization and distribution of both the compound library and automated screening infrastructure.

Scalable synthesis and expansion of compound collections for screening has been a problem since the inception of HTS discovery platforms. Innovations in parallel solid-phase synthesis emerged in the 1990s as HTS platforms exhausted available supplies (routinely ∼100000 compounds). The astonishing efficiency of split-and-pool synthesis easily yielded numerically large chemical libraries. Subsequent screening experiments demonstrated that these “one-bead-one-compound” (OBOC) libraries could be deployed in an array of sophisticated biochemical activity and cellular assays, but a new bottleneck surfaced: hit structure elucidation. Hit structure elucidation by direct mass spectral analysis was still manual and low-throughput, inspiring the encoding of combinatorial synthesis in more analytically tractable chemotypes (e.g., peptides, mass tags). Nucleic acids were proposed and implemented for encoding as well, but state-of-the-art DNA sequencing in the pregenome era was as tedious and low-throughput as any of the other chemical analyses.^8,9^

**Figure 1:**
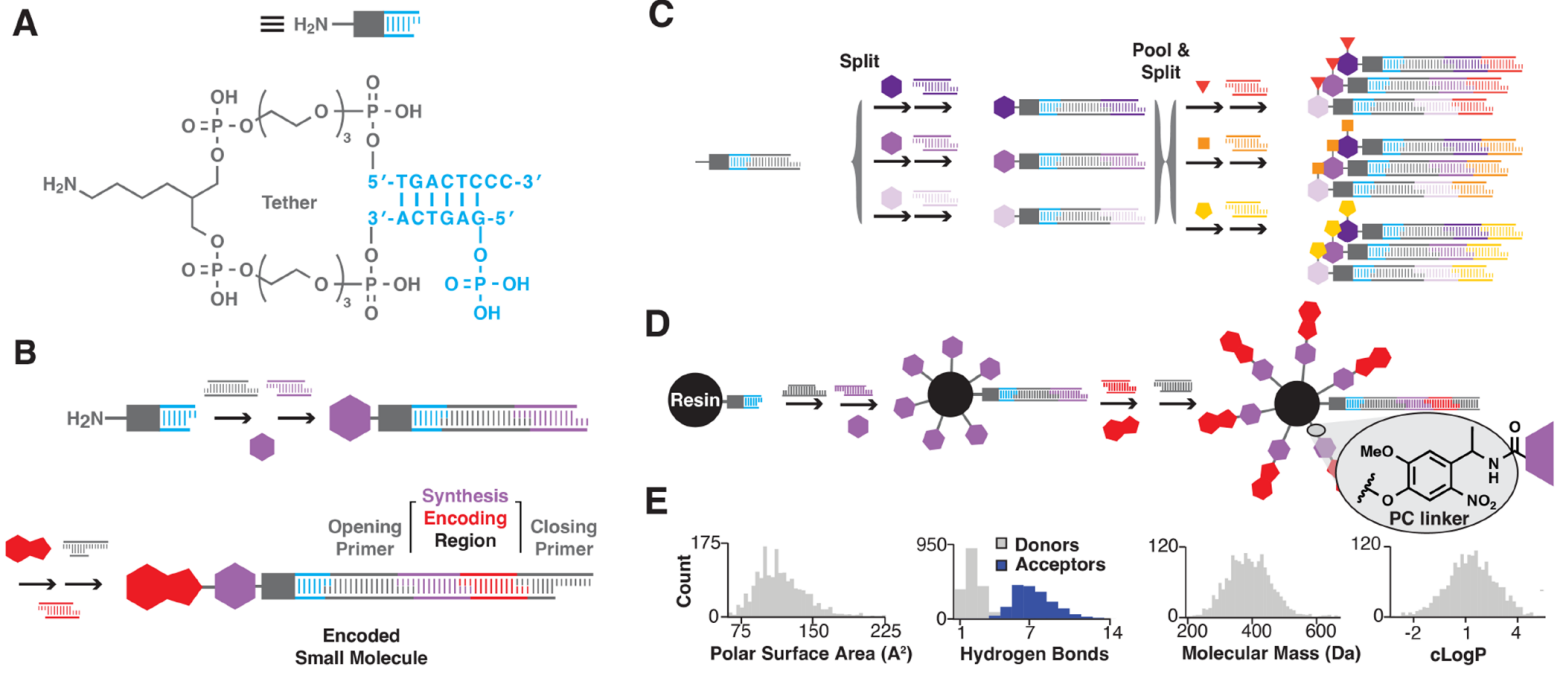
DEL synthesis tactics and example property distribution. (A) The DNA “headpiece” contains a PEG linker that covalently tethers double-stranded DNA containing a dinucleotide overhang for enzymatic ligation and a primary amine for chemical synthesis. (B) DNA-encoded synthesis comprises alternating building block coupling and encoding DNA ligation reactions. DNA is assembled stepwise, and the tag contains two primer binding sites (opening and closing) shared among all members. (C) Encoded libraries are prepared using split-and-pool tactics. One split with three building blocks yields three library members, a second split yields nine members, and so on. (D) Encoded synthesis performed on a solid support furnishes polyvalent beads that each display many copies of a single DEL member and its associated DNA encoding sequence. Library members are coupled to the solid support via an *o*-nitroveratryl photocleavable (PC) linker. (E) Physicochemical property distributions from a 2-cycle combinatorial library, consisting of an amine acylated with a carboxylic acid, fall within the rule of 5. Adapted with permission from refs (1) and (15). Copyrights 2019 and 2021 American Chemical Society.

The rise and widespread adoption of genome-scale sequencing technology singularly resurrected combinatorial chemistry by summarily eliminating the hit structure elucidation bottleneck. DNA-encoded library (DEL) technology enabled ligand discovery from numerically large libraries of combinatorially synthesized small molecules that were associated with DNA-encoding tags after affinity selection using high-throughput DNA sequencing.^10^ The DNA-encoded chemical synthesis and selection workflow^11,12^ begins with a DNA headpiece (Figure 1A). DNA tags, which encode the synthetic route to each library member, are ligated enzymatically prior to coupling each chemical building block (Figure 1B). Encoded couplings are parallelized using split-and-pool tactics to generate the encoded combinatorial library (Figure 1C). DELs are screened via affinity selection. The DEL is incubated with an immobilized protein target, unbound members are washed away, and the bound fraction DNA encoding tags are sequenced to elucidate all hit structures. DELs of astronomical diversity (>10^8^) are now accessible via diverse DNA-compatible bond construction strategies,^13−15^ and DEL screens have been successfully deployed against numerous targets with some DEL-derived ligands now in clinical trials.^16−18^

DEL technology solves the chemical synthesis and analysis bottleneck, but DEL screens are limited to binding affinity selection. As DELs are inherently complex mixtures, they are not suitable for the wider array of library screening assays, such as biochemical activity assays or phenotypic cellular assays. To circumvent this limitation, our group integrated DEL principles with the OBOC combinatorial synthesis approach, wherein bifunctional synthesis resin is subjected to the same DNA-encoded split-and-pool cycles to yield OBOC DELs (Figure 1D).^19^ Furthermore, we showed that substoichiometric bead surface functionalization with DNA encoding sites still yielded adequately amplifiable DNA, even after multiple rounds of chemical synthesis. Implementing a cleavable linker (e.g., photocleavable linker) could free the library members from the bead surface, and the liberated DEL members could be designed to exhibit advantageous drug-like physicochemical properties (Figure 1E). Having rendered the compound library distributable (and even disposable), screening such liberated DEL members would require an equally scalable approach.

## 2 Next-Generation Molecular Screening

Molecular screening for drug discovery was one of two applications that microfluidic miniaturization was destined to revolutionize in the 1990s. While nanoliter (or smaller) assay volumes and microfluidic sample handling automation could dramatically reduce many aspects of the HTS footprint, a compound library format that could scalably interface with this microfluidic world did not exist. A solution resided in OBOC technology.

### 2.1 System Engineering and Assay Miniaturization

**Figure 2:**
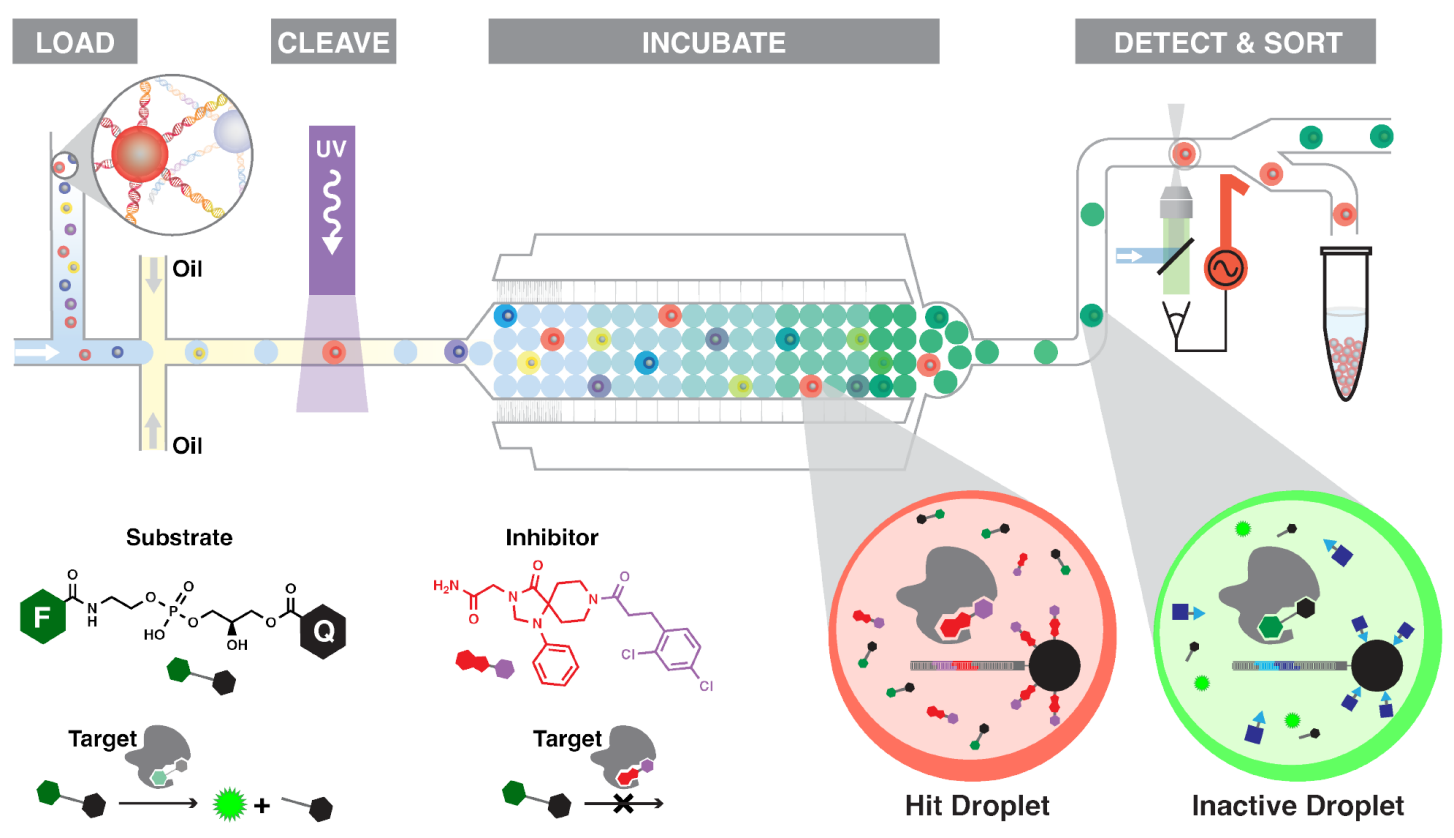
Microfluidic activity-based DEL screening. A microfluidic device encapsulates DEL beads in droplets of assay reagent. UV irradiation cleaves the DEL member from the bead, and an incubator slows the flow to allow the assay signal to develop. Laser-induced fluorescence detection permits high-sensitivity identification of hit droplets, which exhibit attenuated signal and are sorted into the “hit” outlet (top). Droplets contain an enzyme target, a DEL member, and a fluorogenic probe. The example probe is a phosphodiesterase substrate, which contains a fluorophore (F) and quencher (Q) separated by a phosphoglycerol junction. Enzymatic cleavage of the phosphodiester bond increases droplet fluorescence. Hit DEL bead-containing droplets (red) exhibit low fluorescence signal. Inactive DEL bead-containing droplets (green) exhibit high fluorescence (bottom). Adapted with permission from ref (23). Copyright 2017 American Chemical Society.

Building on two decades of vigorous technology development in combinatorial synthesis and microfluidic miniaturization, we set about integrating numerous advances into a next-generation DEL screening platform. Driving the inception of this platform was a simple calculation: a relatively small synthesis resin particle (10 μm diameter, 100 fmol loading capacity) that is encapsulated in a 100 pL droplet can generate highly concentrated solutions (1 mM) of the bead cargo were all of it to be released into the droplet volume. By encapsulating the beads in droplets of the activity assay reagent, such as an enzyme–substrate biochemical assay, one could discriminate between the presence of an active inhibitor and an inactive compound by measuring the droplet fluorescence (Figure 2). The adaptation of known microfluidic components and invention of new parts specifically tailored for bead handling yielded a fully integrated device capable of loading library beads into microfluidic droplets for activity assay,^20^ photochemical cleavage of library member into the droplet in flow,^21^ droplet incubation,^22^ and high-speed laser-induced fluorescence detection of the assay result to inform droplet sorting.^23^

A major early concern was whether this new compound screening approach would be compatible with the panoply of assay types and detection modalities deployed in routine HTS campaigns and with commensurate statistical power. To qualify all assays, we conceived a rigorous approach to statistical assay development in droplets that mirrored the requirements of HTS experiments within the MLP Center Network, as described in the NIH’s Assay Guidance Manual (AGM).^24^ The AGM called our attention to the statistical assay quality score, *Z*′, which is calculated according to eq 1
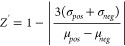
1where μ_*neg*_ and μ_*pos*_ are the mean assay signals without (*neg*) and with (*pos*) positive control compound and σ_*neg*_ and σ_*pos*_ are the corresponding standard deviations. For a statically perfect assay, *Z*′ = 1.^25^ An assay that is acceptable for screening is *Z*′ > 0.5. Although this analysis is usually conducted in microplates, it can be used to evaluate droplet-scale assays through flow injection analysis. Droplets containing assay alone (*neg*) or with a positive control compound (*pos*) are analyzed, generating two populations of assay signals that can be fitted with Gaussian distributions to obtain all four parameters needed to calculate *Z*′ for the droplet-scale assay.

**Figure 3:**
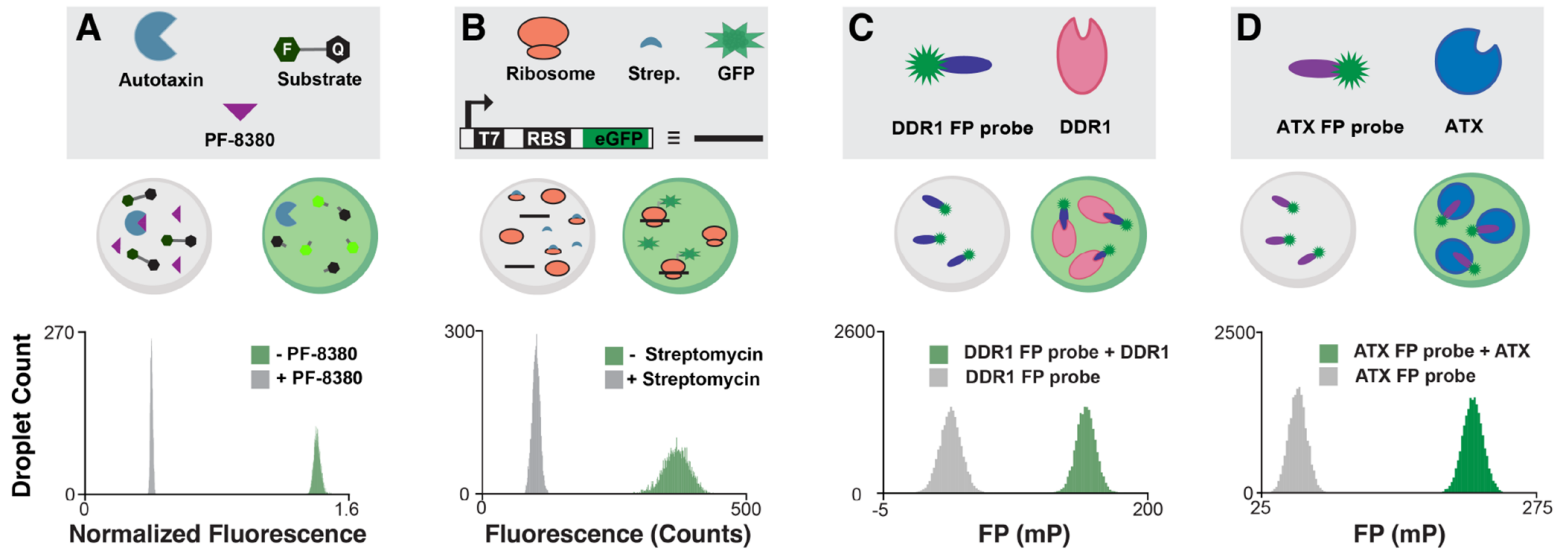
Droplet-scale assay development for DEL screening. For each assay, negative and positive control condition droplets are analyzed. The mean droplet laser-induced fluorescence and variance are extracted for each condition to calculate the assay quality, *Z*′ (*Z*′ > 0.5 indicates suitability for screening). (A) In a biochemical autotaxin (ATX) activity assay, droplets containing a fluorogenic substrate and enzyme are fluorescent; positive control ATX inhibitor PF-8380 attenuates droplet fluorescence (*Z*′ = 0.88). (B) In a bacterial translation activity assay, control droplets fluoresce upon GFP translation. Droplets containing translation inhibitor streptomycin have low fluorescence (*Z*′ = 0.64). (C, D) In a fluorescence polarization (FP) assay of ligand binding, droplets with unbound FP probes exhibit low FP, while droplets with bound probes have high FP. Droplet-scale assay quality was suitable for both a DDR1 and ATX binding assay (*Z*′ = 0.56 and 0.67, respectively). Adapted with permission from refs (2) and (22). Copyright 2017, 2020 American Chemical Society.

Using this approach, robust assays were developed and successfully miniaturized to the droplet scale for diverse biochemical targets. Hydrolase targets included HIV aspartyl protease,^20^ cathepsin D aspartyl protease,^23^ and the phosphodiesterase autotaxin (ATX, Figure 3A).^1^ These assays featured a purified enzyme target, which turned over a fluorogenic substrate to yield a fluorescent product. A protein kinase A (PKA) assay and bacterial in vitro translation assay (Figure 3B) were also successfully miniaturized as examples of complex, coupled biochemical systems.^22^ Finally, a laser-induced fluorescence polarization (FP) detection system was constructed and FP-based binding assays were developed and successfully miniaturized for both ATX and discoidin domain receptor 1 (DDR1) kinase (Figure 3C,D).^2^ The FP binding assay signal is an enhancement of FP when a dye-labeled small-molecule FP probe binds a macromolecular target, attenuating the rotational rate of the probe’s dipole and thereby preserving fluorescence emission polarization upon excitation with polarized light.

Droplet microfluidics clearly supported high-quality, picoliter-scale assays of biological activity. Observations during the course of these assay development projects furthermore revealed unanticipated advantages beyond the obvious reduction in reagent consumption. Most notably, the flow chemistry nature of the system dramatically increased the sampling and assay “well” signal uniformity. As a result, assays with substrates that did not exhibit particularly large changes in signal between time 0 and the endpoint were nonetheless sufficiently high quality in droplets (e.g., ATX substrate with 3-fold increase in fluorescence). As the laboratory continued to explore assay development for increasingly diverse targets, we sought more general, scalable assay development strategies, leading to the construction of the FP system. FP probe design and synthesis are generally more straightforward than substrate engineering and further offer opportunities to interrogate targets that do not have readily measured signatures of biochemical activity.

### 2.2 Screening and Statistical Deconvolution

**Figure 4:**
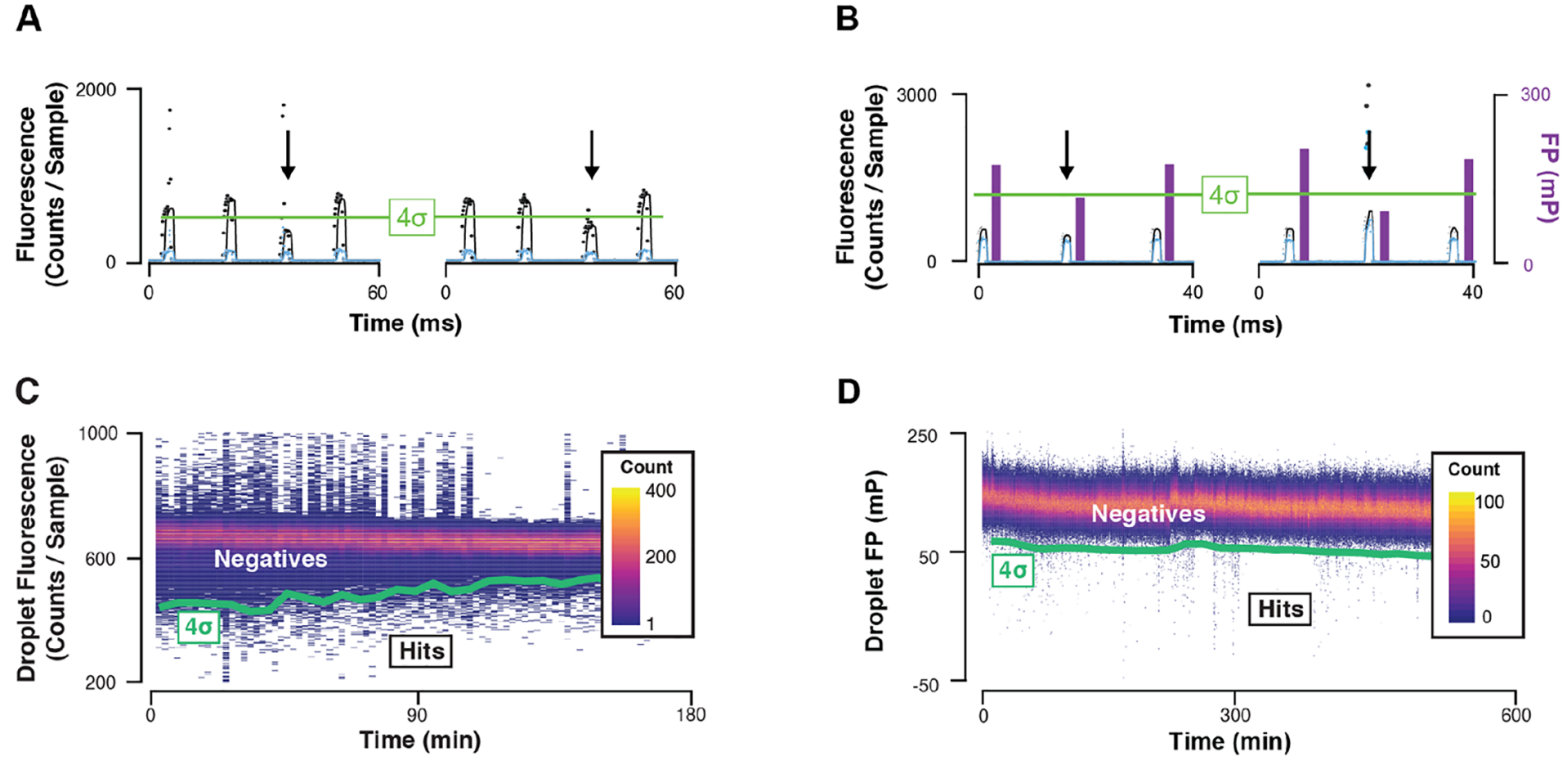
Microfluidic DEL screening data. Droplet signal traces for (A) homogeneous fluorescence emission (ATX) and (B) fluorescence polarization (DDR1) illustrate the concept of high-speed dynamic statistical hypothesis testing. Raw data (points) are smoothed (lines), and each droplet’s profile is reduced to a single value. In the homogeneous fluorescence screen, the assay signal trace is shown in black and droplet markers are shown in cyan. In the fluorescence polarization screen, the plane-polarized emission trace is shown in cyan and the perpendicularly polarized emission trace is shown in black. The mean and standard deviation are calculated for 1000-droplet windows. Droplets with assay signal 4 standard deviations below the mean (4σ line shown in green) are sorted as hits (arrows). Transient heat maps of microfluidic screens for ATX inhibitors (C) and DDR1 competitive ligands (D) using a 67 100-member drug-like DEL illustrate very different hit rates for each target. The 4σ sorting threshold is shown (green). Adapted with permission from refs (1) and (2). Copyright 2019 and 2020 American Chemical Society.

With rigorous assay development workflows in hand, we required equally robust droplet compound screening systems. Screening hits are outliers by definition, and thus we built code to sort hits based on dynamic statistical hypothesis testing. During a library screen, each droplet’s assay signal is detected and compared to a dynamic threshold that is calculated by acquiring both the mean (μ) and standard deviation (σ) of the prior 1000 droplets and setting the instantaneous threshold to μ – 4σ. A droplet with signal < μ – 4σ is a hit and electrokinetically sorted (Figure 4A). This sorting strategy was deployed in microfluidic DEL screens using either the homogeneous fluorescence ATX inhibition assay or the DDR1 FP competition binding assay (Figure 4B). In the ATX screen, inhibitory DEL members attenuated probe hydrolysis, thus low-fluorescence droplets were hits. In the DDR1 competition binding screen, DEL members that competed for FP probe binding to DDR1 attenuated droplet FP, thus low FP droplets were hits.

Microfluidic DEL screening dramatically reduced reagent consumption while enabling complex measurements of DEL member activity by means of droplet compartmentalization. Proof-of-concept screens for ATX inhibitors and DDR1 ligands each explored a 2-cycle drug-like solid-phase DEL (67100 members) and entailed an analysis of ∼10^6^ beads using ∼300 μL assay reagent. Much like a flow cytometry experiment, activity-based microfluidic DEL screening data are acquired in real time and instrument parameters can be adjusted accordingly. For example, the ATX screen hit rate was too high under default conditions; reducing the UV intensity attenuated DEL member photocleavage and therefore concentration in droplets, lowering the hit rate.^1,21^ Default conditions for DDR1 produced a reasonable hit rate at maximal DEL member concentration in agreement with prior DEL affinity selections of ATX and DDR1 wherein the DEL contained fewer DDR1 ligands.^2^ Reasonable hit rates are such that the number of hit beads does not exceed current PCR constraints (∼30000 beads) and is above the anticipated false discovery rate (FDR).

As with any screen, the FDR of microfluidic DEL screening is a function of the negative assay signal standard deviation and the number of reactions that were measured. Under the assumption that the data are normally distributed, eq 2 describes the probability of an observation falling outside one-half of the distribution, the one-tailed *p* value
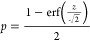
2where erf() is the normal cumulative distribution function and *z* is the number of standard deviations (σ) used for the sort threshold. For example, using the aforementioned 4σ sort threshold, *z* = 4 and *p* = 32 ppm. In other words, for every million droplets screened, 32 would be predicted to be outliers. Thus, if a screen of 10^6^ droplets yielded ≤32 hit droplets, one would conclude that the screen was unproductive for that target and further analysis would not be warranted. Otherwise, the hit collection progresses to sequencing and hit structure deconvolution.

**Figure 5:**
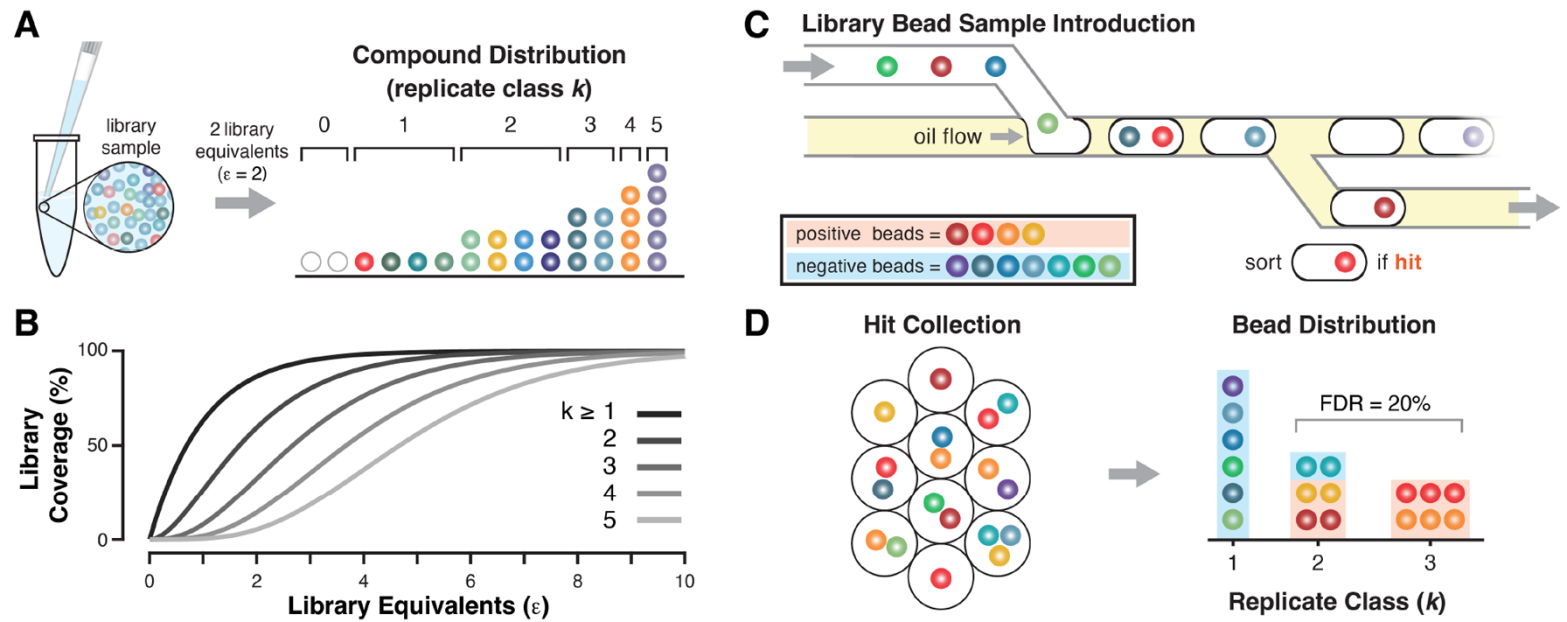
Library sampling and *k*-class deconvolution. (A) An aliquot of DEL contains a distribution of copies of DEL members, dictated by the number of library equivalents (ε, a number of beads equal to the library diversity) sampled. A compound could be present on 1, 2, or *k* replicate beads in the aliquot. The sample distribution is for 2ε, and there is a probability that a library member will not be present in the sample (*k* = 0). (B) The Poisson cumulative distribution function describes the fraction of a library that will be present on at least *k* replicate beads as a function of equivalents sampled. (C) Compartmentalizing DEL beads in microfluidic droplets occasionally results in the coencapsulation of positive beads (warm hues) with negative beads (cool hues). We assume that the device sorts droplets containing at least one positive bead with high fidelity. (D) Hit collection beads are harvested and aggregated by *k* class. Only authentic hits are systematically overrepresented in hit droplets and can be readily distinguished via *k* class from inactive DEL beads that were randomly coencapsulated with hit beads. The FDR drops exponentially as lower *k*-class hits are omitted from consideration (here FDR = 20% at *k* ≥ 1 and 0% at *k* ≥ 2). Adapted with permission from ref (4). Copyright 2017 American Chemical Society.

Hit deconvolution, the process of relating hit collection DNA encoding tags to chemical structures, is also a statistically rigorous process that relates library sampling to each hit’s authenticity. The number of beads in the DEL aliquot used for screening determines the probabilistic representation of each compound in the screen. Aliquot size is most conveniently measured in equivalents (ε), equal to the library diversity (e.g., 2ε of a 100000-member library is 200000 beads).^4^ Any given library member can be present on multiple beads, a critical property known as the replicate class, or *k* class (Figure 5A). As the screening aliquot size increases, the fraction of the DEL that is present in at least *k* copies, or % coverage at *k*, increases and is described by the cumulative Poisson distribution function (Figure 5B). The presence of multiple copies of each compound is critical for addressing the aforementioned FDR and the probability that an inactive bead might be encapsulated with a hit bead during microfluidic screening (Figure 5C). However, if the instrument reliably sorts all droplets containing at least one hit bead, then the hits can be aggregated and analyzed by *k* class. Only authentic hits will be systematically enriched in the hit collection; coencapsulation is random. Triaging low-*k* class hits can drive the FDR to zero (Figure 5D).^4^

### 2.3 Validation and Synthesis

After filtering hit beads by *k* class, the encoding regions are decoded to molecular structure for further analysis and prioritization of follow-up syntheses for validation. The *k*-class filter ensures that all structures considered in the hit collection have as low an FDR as possible. Due to the noise of Poisson sampling and chemical synthesis,^4,26^ further stratification by *k* class beyond the triage is not warranted. Analogous to affinity DEL hit analysis, activity-based hits are visualized by building block identity in each cycle. Building block conservation among multiple high-*k* hits increases the priority of the hit or series for synthesis and follow-up. For example, dichlorophenyl-containing building blocks were seen in numerous high-*k* ATX screening hits in both chemistry cycles 1 and 2, a generally important feature of ATX inhibitors.^1^ Similarly, the high-*k* DDR1 screening hit collection was enriched in a piperazinyl quinazolinone amino acid in cycle 1 and an elaborated azaindole in cycle 2, both known receptor tyrosine kinase ligands.^2^

**Figure 6:**
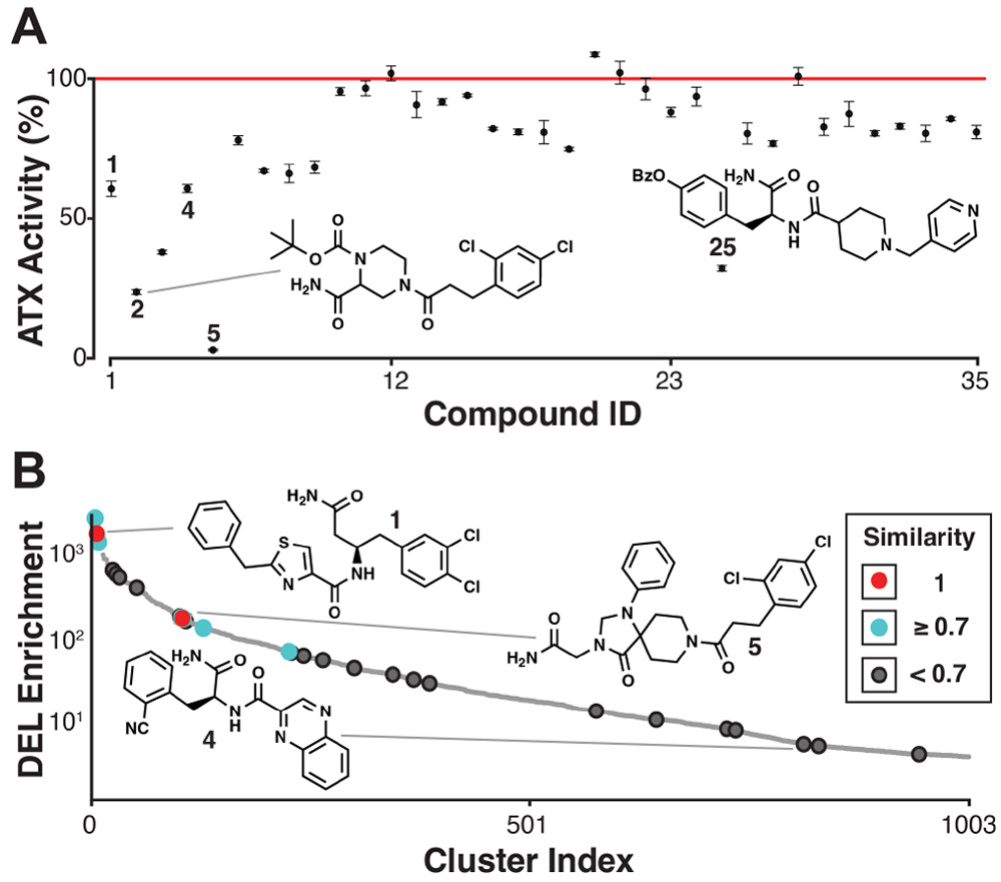
Hit validation and comparison with affinity DEL. (A) High-*k*-class ATX inhibitor screening hits were prepared individually via solid-phase synthesis, photocleaved, and assayed for activity. Hits 1–5 all exhibited IC_50_ ≤ 10 μM. (B) Activity-based DEL screening hits were assigned an affinity DEL screening hit cluster index according to the most similar affinity DEL hit cluster representative (Tanimoto similarity). Some activity-based hits were identical to the affinity DEL hit cluster representatives (1, 5; red) while others were very similar (2; cyan). Activity-based screening identified all of the top-enriched affinity DEL clusters that were present in the solid-phase DEL. Some activity-based hits (1, 25) correlated with high-affinity DEL enrichment, while others were active but did not as appreciably enrich in the affinity selection (4).^1^ Adapted with permission from ref (1). Copyright 2019 American Chemical Society.

Building block enrichment guides subsequent decisions to synthesize hits at scale for validation. Solid-phase synthesis is fairly amenable to parallelization and scale-up to access meaningful quantities of material for more rigorous chemical analysis. We selected 35 compounds from the ATX screen for synthesis (100 nmol scale) and activity assay of crude photocleavage supernatant, and the majority (20/35) inhibited ATX (<85% activity, Figure 6A). The five most active crude samples were synthesized at scale, purified, and formally evaluated for IC_50_ (all ≤10 μM).^1^ A similar crude photocleavage supernatant analysis of 13 DDR1 competition binding hits also resulted in high validation rates (12/13). Two validated at scale as DDR1 kinase domain ligands (*K*_D_ = 35 and 8 μM).^2^ Both ATX and DDR1 screens used a 67100-member, 2-cycle, solid-phase DEL that was similar to a larger 866000-member, 3-cycle affinity DEL at Roche. The 3-cycle library was used in affinity selections for ATX and DDR1 ligands,^2^ providing a point of comparison for activity-based DEL screening. Affinity DEL prioritizes hits by the rate at which compound encoding tag sequences are enriched in the binding elution fraction, or “enrichment.” Higher enrichment signals target binding, which is a complex function of binding on and off rates. Activity-based ATX DEL screening hits sampled the range of DEL enrichment values (Figure 6B). Activity-based hits showed agreement with affinity hits, but these tended to be higher-mass library members. Lower-mass members tended to agree with lower enrichment, and most activity hits were novel in that they exhibited low similarity even to their most similar affinity DEL cluster.

The ATX and DDR1 screening campaigns in conjunction with prior foundational technology development efforts collectively established proof of concept for a next-generation compound screening platform. Integration of microfluidic components for DEL bead loading, photocleavage, incubation, and sorting^23^ into a complete compound screening circuit eliminates automation overhead while uniquely enabling new modalities for detecting biological activity in DELs. These new measurements could prove useful in identifying DEL members that would otherwise escape detection in an affinity selection. For example, smaller molecules with fast binding off rates (e.g., fragments) may not be detectable even though these hits could be more tractable starting material for medicinal chemistry.^27^ Finally, just as target concentration and multitarget screening are important for prioritizing the massive hit collections of DEL affinity selections,^12,28,29^ there are further parameters to explore in microfluidic screening, such as the UV intensity (i.e., compound concentration)^21^ and statistical sorting threshold, which determines FDR and potentially gates on synthesis fidelity^26^ and photorelease efficiency.

## 3 Opportunities and Outlook

Advances in measurement technologies for DEL screening promise to unlock an abundance of new target spaces and assay modalities. Some target classes are inaccessible to conventional affinity DEL because purification to homogeneity is not possible. Intrinsically disordered proteins and integral membrane proteins are examples. Integral membrane proteins can be investigated via cell surface display,^30−32^ but ligands discovered via this approach do not necessarily exhibit cellular activity, a long-sought assay modality for DEL. Finally, the promise of increasingly complex screening data sets underscores the need for more sophisticated informatics that can prioritize or even predict the best chemical matter for hit generation and lead optimization.

### 3.1 Classically Forbidden Targets

Nucleic acid binding proteins, which play crucial roles in diverse cellular processes, are targets of great interest in drug discovery. For example, transcription factors operate at the bottom of signaling cascades and are likely the most selective target for affecting gene expression, and polymerases are central to replication, making them excellent antiviral targets. Proteins in this class are very difficult to investigate via affinity selection DEL because they have higher affinity for the encoding tags than for the encoded small molecule.^12^ While it is possible to block DNA binding domains with an excess of genomic DNA, microfluidic off-DNA competition binding screening^2^ offers an alternative opportunity to escape this limitation by presenting nucleic acid only at very low concentration and displayed from a solid support where diffusion to the surface is limiting. Multiple studies have shown that highly potent bioactive molecules present on the bead surface do not interact with a protein target in the surrounding droplet in the absence of photocleavage.^2,23^

On-bead screening has also enabled the investigation of nucleic acid targets, such as RNA secondary structures, via DEL. Targeting such RNA structures with small molecules enables transcriptomic target prediction while avoiding the limitations of protein druggability.^33^ These therapeutic strategies require RNA ligands, and a growing body of evidence suggests that RNA targeting may require chemical libraries with physical properties that are not necessarily Ro5-compliant and therefore are unlikely to be well represented in standard compound libraries.^34,35^ DEL technology affords ready access to such diversity and has already proven useful in the identification of ligands of a G quadruplex.^36^ We demonstrated unbiased DEL-on-RNA screening to identify novel small-molecule ligands of RNA from among 300 million possible RNA–DEL interactions. Nonspecific binding was addressed by multiplexing the screen with a differentially labeled off-target RNA. Further affinity selection of the RNA secondary structure library using the DEL-derived ligand statistically implicated its target as the internal loop, 5′ GAG/3′ CCC. Transcriptomic mining identified the loop’s presence in oncogenic primary microRNA pri-miR-27a, and cellular assays confirmed the upregulation of several genes that miR-27a targets, ultimately inhibiting a metastatic phenotype in MDA-MB-231 breast cancer cells.^37^ These first RNA–DEL screening campaigns inspire expanded chemical synthesis, assay, and screening technology development.

### 3.2 Cellular Assay Technology

Phenotypic cellular screening continues to play an outsized role in early drug discovery, particularly for targets of unknown biological function or those that are difficult to produce at scale.^38,39^ The NIH AGM comprehensively catalogs the diversity of cell-based assay types and strategies for assay transfer to HTS operations.^24^ Briefly, cellular assays most commonly entail engineering a cell line to produce a detectable reporter (e.g., luciferase, β-galactosidase, alkaline phosphatase, GFP, and related complementation strategies) in tandem with the expression of a gene of interest or secondary metabolite sensing (e.g., Ca^2+^, cAMP, etc.). Alternatively, high-content imaging can be used to track cell fate as a function of compound treatment with higher granularity, such as the localization of a fluorescent protein-tagged gene of interest. Cell lines can be standard immortalized types or derived from patient tissue. Cellular activity is usually measured on monolayer cultures, but organoid/organ-on-a-chip technologies are attracting increased interest for recapitulating tissue-level functions more closely. Finally, cellular assays inherently measure other pharmacokinetic properties, such as membrane permeability and cytotoxicity. Consequently, there is great interest in adapting these powerful screening tools to DEL.

Interfacing cellular targets with DEL has proven useful, but adapting cellular activity assays with DEL is not yet feasible. An early innovation was affinity-based DEL selections using cell-displayed targets, unlocking proteins that would be otherwise difficult to purify to homogeneity, such as cell-surface receptors NK3, FR, EGFR, and CAXII, and further exploration of this concept has led to intracellular selection technology.^31,32,40^ A common observation from receptor-targeting selections both in cellulo and in vitro is that ligands can be either agonists or antagonists. In other words, selection does not measure cellular activity because DELs are complex mixtures wherein no single library member is present at sufficient concentration to stimulate cellular signaling.

**Figure 7:**
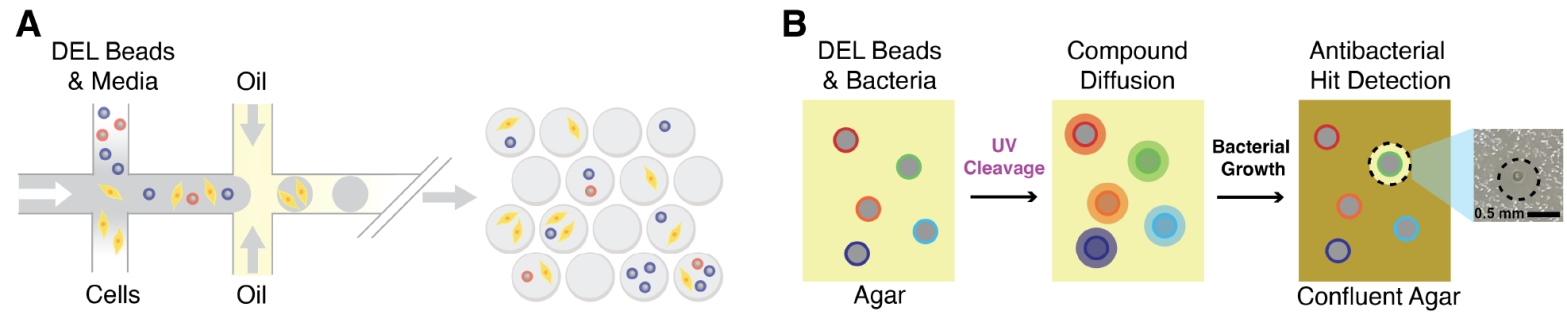
Cellular DEL screening paradigms. (A) Coencapsulation of DEL beads with media and cells. DEL beads and cells are introduced at a mean occupancy of λ = 1. The resulting droplet population contains few one-bead, one-cell pairs (∼10%), compromising assay throughput and eliminating the possibility of a reliable negative control signal for statistical hypothesis testing. (B) Cellular DEL screening using an agar diffusion assay entails depositing DEL beads on agar bacterial lawns, photocleavage, and subsequent library compound diffusion into the agar. Following bacterial growth, hits generate a growth inhibition zone (GIZ) marking them as a hit for manual picking. Adapted with permission from ref (3). Copyright 2021 American Chemical Society.

It is tempting to propose an analogous microfluidic strategy to encapsulate cells and DEL beads; however, coencapsulation introduces significant sampling limitations. Cells and DEL beads are both discrete elements, and droplet sampling creates a double Poisson distribution, wherein a minority of droplets contain both a DEL bead and a cell (Figure 7A). As the cells are the “assay”, not all droplets contain a negative control signal, which is necessary for high-power dynamic statistical hypothesis testing-based sorting. Further complicating matters, cellular signaling variance is inherently higher than biochemical assay signal variance. These two factors cast a pall over the prospect of a direct port of microfluidic screening capabilities to cell-based screening. However, recent innovations in droplet printing show great promise in obviating the Poisson limitations of random droplet loading,^41,42^ and an investigation of alternative cell culture strategies is underway, also in an effort to eliminate these liabilities while sustaining the screening throughput needed to address DEL-scale diversity.

Antibiotic discovery is another significant area of opportunity for DEL technology. The rising rate of antibiotic resistance has only intensified while the antibacterial pipeline has dramatically contracted. Comprehensive reviews of industrial antibacterial screening campaigns placed the blame squarely on the compound library content: strict adherence to the rule of 5 left compound libraries largely bereft of chemical matter exhibiting physicochemical properties that are commonly observed in antibacterials.^43,44^ Combinatorial library synthesis could enable the exploration of these novel chemical spaces, especially when paired with direct assays of bacterial cytotoxicity via bead diffusion or lawn assays, which identify hits via growth inhibition zones (GIZs).^45−47^ We demonstrated that these cellular screening concepts could be employed using solid-phase DELs in whole-cell *E. coli* and *B. subtilis* lawn assays. Single 160 μm beads were distributed on agar plates and irradiated to photocleave DEL members and initiate diffusion into the agar. Bacteria proliferated in a lawn and GIZs emerged around antibacterial DEL beads, marking them as hits for manual picking (Figure 7B). The screen yielded multiple cell-active hits, some of which recapitulated known fluoroquinolone structure–activity relationships.^3^ However, manual bead picking limited library diversity. Innovation in library bead picking automation or analysis in flow paired with culture miniaturization and high-throughput viability sensing could form the basis of a powerful next-generation antibacterial discovery platform.

### 3.3 Computational Intervention

DEL technology enables in theory and in practice the synthesis of vast and novel chemical spaces.^48^ Empirical analysis has concluded that the size and complexity of DELs do not necessarily correlate with an increased hit rate^49^ and that it is pointless to include all available building blocks, especially those containing structural flags.^50^ These studies posit that smaller, tailored libraries using simple and robust bond construction may be more efficient and fruitful. Product-based or building block-based modeling of DELs can maximize the diversity of compounds within a focused and application-appropriate property space. Moreover, machine learning (ML) can now predict synthesis quality through sparse samplings of building block coupling efficiencies to aid DEL design.^51,52^ These capabilities will become increasingly important as DEL is used to expand the target scope by exploring chemical space beyond the rule of 5 (bR05), where the prediction of synthesis efficiency and many physicochemical properties becomes difficult or impossible.

In addition to DEL design, computational approaches are already playing an increased and outsized role in DEL screening hit selection, follow-up hit expansion, and hit-to-lead activities. The inherently structured data of DEL screening output makes it highly amenable to training ML models for identifying probable hits during virtual screens or analyzing screening data for SAR using monomer or synthon enrichment.^53^ For example, a graph convolutional neural network performed with strong generality, predicting hits in new chemical spaces for diverse protein targets, including sEH, ERα, and c-KIT.^54^ Including modeling in DEL preparation workflows could also enhance hit rates and denoise data in difficult areas of discovery, such as antibacterials and membrane permeation among others. Analogous to ML-guided synthesis prediction, ML-guided SAR and hit projection could be invaluable for downsampling bRo5 encoded combinatorial library screening hits to structures that are more amenable to further medicinal chemistry optimization.

## 4 Closing Remarks

Scaling considerations guided the conception of a next-generation miniaturized compound screening platform, and as we think about the future, scaling continues to dominate our technology development hypotheses. The implementation of DEL principles and miniaturization of the compound library format to microscopic beads formally democratize and commoditize chemical diversity. DELs are now consumables, much as primers, chips, and terminator mixes are consumables for DNA sequencing. Similarly, microfluidic DEL screening prototypes in our laboratory and in others’ laboratories pave the way for commercial instrumentation that can distribute screening, just as microfluidic instruments distributed DNA sequencing operations globally. However, assay development is the elephant in the room. DNA sequencing is scalable because sequencers run the same assay regardless of nucleic acid input. The same is not true for screening protein function: protein physicochemical properties, structure, and function are highly sequence-dependent and largely unpredictable. Thus, developing a suitable high-throughput assay for any given protein is in itself a project. Proposing such an undertaking for all of the genome-encoded ∼20000 proteins is presently untenable even if all their functions were known and the limitations of druggability were cast aside.

Looking forward, a more general approach to predicting, assaying, and pharmacologically altering biological activity is necessary. Modulating core cellular processes surrounding nucleic acid metabolism is a prime candidate. Selectively perturbing transcription or translation, for example, would move assay development away from mature proteins and toward nucleic acids, which exhibit largely sequence-independent physicochemical properties, highly predictable structure, and well-understood stability. Targeting this layer of metabolism for drug discovery further sidesteps all considerations of mature protein function and thereby the scope of canonical druggability. It is at this intersection of chemical synthesis, technology development, and medicinal chemistry where we see an opportunity to realize the vision of the Human Genome Project and deliver an integrated platform for molecular probe discovery at scale.
